# 88 Does Weight Matter? A Secondary Analysis from ABRUPT

**DOI:** 10.1093/jbcr/irad045.061

**Published:** 2023-05-15

**Authors:** Jeffrey Fine, Sandra Taylor, David Greenhalgh, Robert Cartotto

**Affiliations:** University of California, Davis, Sacramento, California; University of California, Davis, Sacramento, California; University of California, Davis and Shriners Hospital for Children, Northern California, Sacramento, California; Ross Tilley Burn Centre, University of Toronto, Toronto, Ontario; University of California, Davis, Sacramento, California; University of California, Davis, Sacramento, California; University of California, Davis and Shriners Hospital for Children, Northern California, Sacramento, California; Ross Tilley Burn Centre, University of Toronto, Toronto, Ontario; University of California, Davis, Sacramento, California; University of California, Davis, Sacramento, California; University of California, Davis and Shriners Hospital for Children, Northern California, Sacramento, California; Ross Tilley Burn Centre, University of Toronto, Toronto, Ontario; University of California, Davis, Sacramento, California; University of California, Davis, Sacramento, California; University of California, Davis and Shriners Hospital for Children, Northern California, Sacramento, California; Ross Tilley Burn Centre, University of Toronto, Toronto, Ontario

## Abstract

**Introduction:**

Burn shock resuscitation fluids should be titrated to achieve a urine output (UOP) of 0.5 to 1 mL/kg/hr. It is unknown whether this UOP goal should be indexed to actual body weight (ABW) or predicted body weight (PBW). The purpose of this study was to evaluate differences related to ABW and PBW-indexed UOP using data from ABRUPT.

**Methods:**

We queried the database from ABRUPT, which was a multicenter prospective observational study of acute fluid resuscitation practices involving 379 adults treated at 21 burn centers between 4/2017 and 6/2020. Values are presented as the mean ± SD or median (IQR) as appropriate.

**Results:**

There were 379 subjects (age 46 ± 15.9 yrs, %TBSA burn 31.6 (18), 12.7% with inhalation injury). ABW (N=379) was 90 ± 24.8 kg and PBW (N=375) was 69.5 ± 10.4 kg. ABW exceeded PBW in 84% of subjects. In the 1^st^ 24 h post burn, UOP was 0.87 (0.51) mL/kg ABW/h compared to 1.1 (0.6) mL/kg PBW/h (Figure). Total UOP in the 1^st^ 24 h did not differ between body mass index (BMI) categories. Neither ABW UOP nor PBW UOP was predictive of the decision to initiate 5% albumin (OR and 95% CIs 0.914 [0.754, 1.109] and 0.882 [0.687, 1.133], respectively). We compared subjects with a mean hourly UOP < 0.5 mL/kg/h based on ABW but still > 0.5 mL/kg/h based on PBW (N=61) with subjects whose mean hourly UOP remained > 0.5 mL/kg/h based on ABW (N=283). Subjects whose UOP was < 0.5 mL/kg ABW/h but > 0.5 mL/kg PBW/h were significantly older than subjects whose UOP was > 0.5 mL/kg ABW/h (51.2 [15.6] Vs 44.4 [15.4] yrs, p=0.002), but otherwise we found no significant differences within the 1^st^ 24 hours between these two groups in heart rate, blood pressure, peak lactate, creatinine, total fluids administered, or SOFA score. There were no significant differences in SOFA at 48 and 96 hours, ventilation days, need for dialysis in the 1^st^ 96 hours, or survival.

**Conclusions:**

ABW in a large majority of the ABRUPT population was greater than PBW. Consequently UOP was numerically lower when indexed to ABW than when indexed to PBW. Patients whose UOP was < 0.5 mL/kg ABW /h but > 0.5 mL/kg PBW/h did not appear to have any worse outcomes than those whose UOP remained > 0.5 mL/kg ABW/h.

**Applicability of Research to Practice:**

This study generates a hypothesis and a question as to whether titration based on PBW UOP rather than ABW might result in more aggressive titration and administration of less resuscitation fluid, since PBW-indexed UOP ‘looks’ higher. While this can only be answered in a randomized comparison between the two approaches, our study did not identify any adverse outcomes in patients who had a UOP < 0.5 mL/kg ABW/h but > 0.5 mL/kg PBW/h, which might indicate that titration to > 0.5 mL/kg PBW is feasible.

